# Fluorinated Human Serum Albumin as Potential ^19^F Magnetic Resonance Imaging Probe

**DOI:** 10.3390/molecules28041695

**Published:** 2023-02-10

**Authors:** Dmitry E. Mitin, Alexey S. Chubarov

**Affiliations:** 1 Institute of Chemical Biology and Fundamental Medicine SB RAS, 630090 Novosibirsk, Russia; 2 Faculty of Natural sciences, Novosibirsk State University, 630090 Novosibirsk, Russia

**Keywords:** human serum albumin, N-homocysteinylation, ^19^F-NMR, magnetic resonance imaging probes, fluorinated homocysteine thiolactone, octafluorotoluene, perfluoro-m-xylene

## Abstract

Fluorinated human serum albumin conjugates were prepared and tested as potential metal-free probes for ^19^F magnetic resonance imaging (MRI). Each protein molecule was modified by several fluorine-containing compounds via the N-substituted natural acylating reagent homocysteine thiolactone. Albumin conjugates retain the protein’s physical and biological properties, such as its 3D dimensional structure, aggregation ability, good solubility, proteolysis efficiency, biocompatibility, and low cytotoxicity. A dual-labeled with cyanine 7 fluorescence dye and fluorine reporter group albumin were synthesized for simultaneous fluorescence imaging and ^19^F MRI. The preliminary in vitro studies show the prospects of albumin carriers for multimodal imaging.

## 1. Introduction

Magnetic resonance imaging (MRI) is one of the most powerful noninvasive imaging techniques. ^1^H MRI allows real-time visualization of tissues, physiological processes, the diagnosis of diseases, monitoring the response to therapy and various biological events, and tracking pharmaceutical substances. MRI has unlimited diagnostic depth, high resolution, and excellent contrast abilities. However, in many cases, MRI requires a specific contrast agent (CA) to enhance the difference between the target tissue and the background [1,2,3,4]. The CA for ^1^H MRI is detected indirectly by their effect on the spin relaxation of water protons. Paramagnetic metal complexes of Gd and Mn, and iron oxide nanoparticles are among the primary-used CA [1,3,4,5,6,7]. However, recent concerns regarding low stability and potential toxicity of Gd-based and Fe_3_O_4_ nanoparticles CAs make their clinical use challenging [8,9,10,11,12,13,14]. Therefore, new metal-free technologies were developed [15]. Organic radical CAs have the same contrast mechanism as Gd chelates and are assembled from highly biocompatible species [16,17,18]. However, rapid radical reduction reactions in vivo and low relaxivity, both limit area progress. The earlier stages’ results of the proposed construction show high perspectives for these CAs in the future [16,19]. Another approach is a multinuclear MRI called nonproton MRI [20]. The use of multinuclear MRI has great potential in clinics due to the absence of tissue background in most cases [20]. Magnetically abundant nuclei such as ^19^F and ^31^P are the most popular for drug delivery and metabolism studies [20,21]. The ^19^F consists of 100% naturally occurring fluorine. It has a nuclear spin of 1/2 and a high gyromagnetic ratio, providing the most sensitive heteronuclear NMR measurements. Moreover, no background fluorine NMR signal in a human organism with optimal relaxation time possesses the ability to register ^19^F-containing molecules as “fire in the darkness.” Indeed, the ^19^F NMR chemical shifts span a high range of about ~300 ppm, which may be useful for multiple ^19^F imaging [22]. In recent years, ^19^F NMR/MRI has been used to monitor the biodegradation, biodistribution, and pharmacokinetics of fluorinated drugs, binding studies involving protein-DNA complexes, small molecule interactions with proteins, etc. [23,24,25,26,27,28,29,30]. Structural analysis of protein folding, DNA and RNA secondary structures, various macromolecule complexes, and their changes was performed using ^19^F NMR [28,30]. Recently, ^19^F electron nuclear double resonance (ENDOR) spectroscopy was evaluated for distance measurement between a spin probe and a fluorine label [31,32,33]. Fluorine-based tracer construction can be engineered to contain polymers [34,35] and nanoparticles [29,30,35,36,37,38] for cancer [35,39,40], Alzheimer’s disease, amyloid-β [41,42,43], neuro disorders [44], inflammation [45], and other various pathological conditions [26]. Improvements in ^19^F MRI equipment combined with the development of novel, sensitive probes will decrease the global disease burden.

Herein, we developed human serum albumin (HSA) based construction as a potential dual-modal CA for ^19^F MRI and fluorescence imaging. HSA is a major multifunctional protein in blood plasma [46,47,48]. It plays an important role in hormones, fatty acids, metal cations, and pharmaceutical transport [47,49,50,51,52,53,54]. HSA has attracted attention for therapy and diagnostics [10,49,50,51,52,53,54]. Albumin provides important features as a carrier platform: (1) high biocompatibility and lack of toxicity and immunogenicity; (2) a long half-life of 20 days in the bloodstream; (3) tumor accumulation by enhanced permeability and retention (EPR-effect) of macromolecules and receptor interaction; (4) robustness toward chemical modification; and (5) easily controlled surface chemistry [10,33,49,50,51,52,53,54,55,56,57]. Due to its properties, HSA has been utilized as a carrier for MRI probes and cancer treatment [53,55,56,57,58,59,60,61,62,63,64].

The maleimide-containing fluorescent dye Cy7 was chosen for Cys34 selective labeling. Conjugation or binding of drugs and dyes to albumin is a well-established technology [10,47,49,52,53,54]. Only one Cys34 of HSA has the free thiol group, which can be used for site-specific protein modification. On the other hand, the fluorine label should be multiplied to achieve enough ^19^F MRI sensitivity. We have suggested using a natural posttranslational modification of HSA to label lysine residues in proteins [65]. Homocysteine thiolactone (HTL) is a site-specific acylation compound [16,63,65] that can be used for the covalent conjugation of Lys residues, called N-homocysteinylation. The reaction involves only hyper-reactive Lys residues [16,63]. The predominant site is Lys525, and it has one of the lowest p*K*_a_ among the albumin residues. Thiolactone chemistry provides a powerful tool to prepare fluorine- and spin-labeled albumin-based multimodal imaging probes and therapeutic agents [16,62,63,64]. HTL can be *N*-acylated by a fluorinated compound to produce the reagent for protein labeling. In the present work, we present the synthesis of N-fluorinated HTL compounds for HSA N-homocysteinylation. The acylation reagent synthesis, mechanism of formation, and kinetics studies were investigated. Two types of fluorinated albumin were tested as potential metal-free probes for ^19^F MRI. Dually labeled albumin with fluorescence dye (Cy5 or Cy7) and fluorine were synthesized to obtain a multimodal imaging probe. The conjugates were characterized by mass spectrometry (MALDI ToF MS) and ^19^F NMR. The biocompatibility was checked by sodium dodecyl sulfate-polyacrylamide gel electrophoresis (SDS-PAGE), circular dichroism (CD) spectroscopy, dynamic light scattering (DLS), and primary-used cytotoxicity test (MTT test) [66,67]. The trypsin proteolytic kinetics were applied to show the conjugates’ biodegradability in pseudo-tumor conditions. The results indicate the promise of fluorinated albumin to serve as a probe for ^19^F MRI.

## 2. Results and Discussion

### 2.1. Synthesis of N-Fluorinated Homocysteine Thiolactone Derivatives

**Figure 1:**
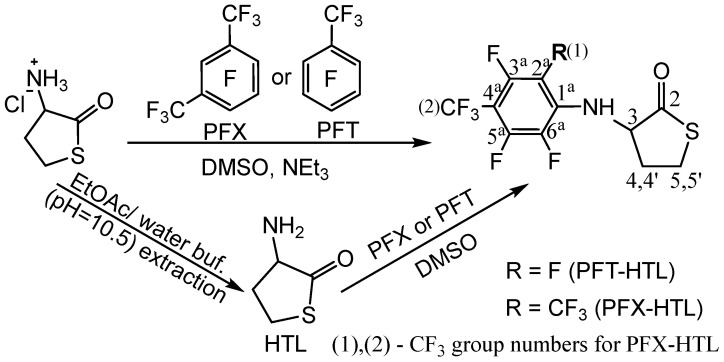
Synthesis of the fluorinated *N*-substituted HTL derivatives.

Homocysteine thiolactone (HTL) is a natural active cyclic thioester of nonprotein homocysteine amino acid (Hcy) [65]. HTL has a strained five-membered cycle, which may react with various nucleophiles [65,68]. HTL is a powerful synthetic tool for functional material production and protein labeling [16,69,70]. The amino group of HTL easily reacts with electrophilic reagents, which can be used for chemical modification. The synthesis of fluorinated HTL was performed according to one- or two-step procedures (Figure 1).

Nucleophilic aromatic substitution (S_N_Ar) of fluorine in perfluoroarenes was used for stable bond formation with the HTL amino group. Dimethylsulfoxide (DMSO) solvent was utilized for HTL derivative synthesis. DMSO is an important solvent that dissolves both polar and nonpolar compounds and greatly increases the rate of nucleophilic substitution reactions. It exhibits several capabilities, such as high biocompatibility (nontoxic class 3 by the FDA), anti-inflammatory and bacteriostatic functions, and an antioxidant [71,72]. DMSO is widely used in life sciences and as an auxiliary solvent in the fabrication of polymers and drugs [73].

**Figure 2:**
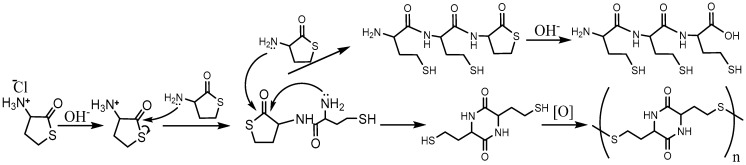
HTL reactions in alkali solution.

The one-stage synthetic pathway consists of an HTL hydrochloride reaction with perfluorotoluene (octafluorotoluene, PFT) or perfluoro-m-xylene (PFX) in the presence of a base (triethylamine) (Figure 1, top). The two-step procedure converts HTL hydrochloride to a free base of HTL with a subsequent reaction with perfluoroarenes (Figure 1, bottom). The free base of HTL synthesis looks simple. Nevertheless, HTL hydrochloride is chemically stable in bulk, and the free base of HTL is extremely reactive. The chemical preparation of an HTL-free base requires a thoughtful synthesis procedure. Due to its electrophilic-nucleophilic character, HTL is susceptible to condensation reactions (Figure 2) [64,74]. Self-condensation of two HTL molecules results in a dipeptide with a thiolactone ring, which may transform into a tripeptide by the intermolecular reaction with the 2,5-diketopiperazine compound (Figure 2). Diketopiperazines are the smallest cyclic peptides that attracted attention due to their ability to mimick peptidic pharmacophoric groups [75]. The wide spectrum of their biological properties is essential for modern medicinal chemistry [75]. The formation of 2,5-diketopiperazine was assumed by Vigneaud V. et al. in 1938 by elemental analysis [74]. The synthesis of fluorinated tripeptide and 2,5-diketopiperazine derivatives of homocysteine was previously confirmed in our laboratory by ^1^H NMR [64]. However, the detailed mechanism of compound formation was beyond the work [64].

The influence of various factors on a free base of HTL synthesis is presented in Table 1. Depending on the reaction time in the alkali solution, a HTL-free base or self-condensation product may be obtained (Table 1). To overcome HTL stability limitations, as low a time as possible in alkali solution and a 0.26 M and lower HTL concentration are required (see Section 3.3.1). However, if the goal of the procedure is to synthesize homocysteine peptides, a higher concentration of HTL and a 10–20 min time frame should be used. 

High-time (30 min) in an alkali solution results in homocysteine 2,5-diketopiperazine disulfide polymer precipitation (Figure 2), as demonstrated by polymer reduction with dithiothreitol (DTT) and ^1^H NMR analysis. By comparing different organic solvents and considering their boiling point, miscibility with water, toxicity, and hazardousness, ethyl acetate turned out to be the solvent of choice. The amino group of HTL has extremely low p*K*_a_ of 6.67 [76], 7.1 [77], and herein 6.56 ± 0.03 (25 °C) and 6.40 ± 0.02 (37 °C) (Supplementary Materials). Due to the almost neutral HTL charge in alkali solution, HTL is easily and with good efficiency (87%) extracted in ethyl acetate solution. Possible side reactions like HTL self-condensation could be suppressed by performing the organic solvent evaporation at as low a temperature as possible (self-cooling). The HTL was obtained at a moderate rate of 96%. In the case of a 0.26 M HTL concentration and 10 min in alkali with subsequent extraction and evaporation, the HTL yield is reduced. The major reaction byproduct is 2,5-diketopiperazine of homocysteine. Using a higher HTL concentration of 0.4 M, homocysteine tripeptide can be synthesized in greater quantities. It should be mentioned that the free base of HTL is not stable even when the temperature is below zero (Table 1). This compound should be used as quickly as possible for further synthesis.

**Figure 3:**
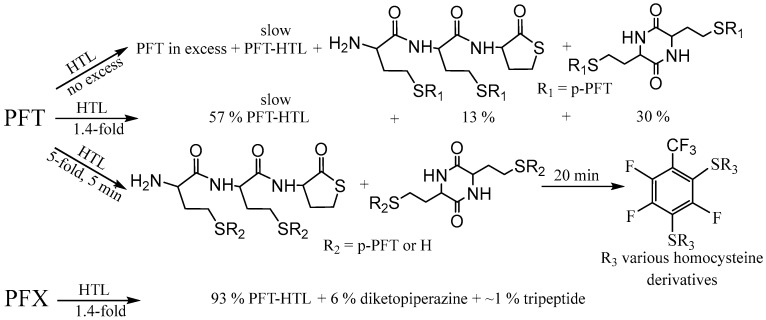
HTL-free base reaction with PFT and PFX in various ratios. The PFT:HTL ratios are 1:1 (line 1), 1:1.4 (line 2), and 1:5 (line 3). Line 4 PFX:HTL ratio is 1:1.4.

The intrinsic instability of the HTL-free base requires highly reactive electrophiles for efficient reaction with the amino group; examples include acid halides, activated esters, and anhydrides [58,69]. Herein, we show the possibility of obtaining N-substituted derivatives of perfluoroarene. The fluorinated thiolactones were derived from the corresponding perfulorotoluene (PFT), perfluoro-m-xylene (PFX), perfluoro-m-xylene and perfluoro-o-xylene mixture. The S_N_Ar is generally held to proceed either stepwise (Meisenheimer complexes) or by a concerted mechanism, which depends on the structural features of the reactants [78,79]. In a perfluoropolyaromatic compound, substitution may occur at one position preferentially. According to experimental data and theoretical calculations [80], the main S_N_Ar sites for PFT and PFX are ortho- and para-positions towards the CF_3_ group [79,81]. Previously, we found that the reaction of HTL with PFT occurs in para position. Using a 1.4-fold excess of HTL, the PFT-HTL was found to be a major product with a 57% yield (Figure 3). In a side reaction, S-fluorinated 2,5-diketopiperazine and tripeptide derivatives were obtained (Figure 3 and Figure S1) [64]. Nevertheless, the initial HTL-free base doesn’t contain a sufficient amount of self-condensation products to indicate their synthesis in DMSO solution during the reaction. Moreover, the amino group of HTL reacts with PFT much slower than the SH group of homocysteine derivatives, as shown by ^19^F NMR. About 5 min is enough for SH nucleophiles to yield a S_N_Ar reaction, in comparison toNH_2_ that requires several hours. High excess of HTL results in a PFT quick substitution in para- and then ortho-position, which can easily be observed by CF_3_ signal changes from triplet to duplet state by ^19^F NMR. Instead of PFT, the PFX compound reacts with HTL free base much quicker, with a PFX-HTL yield of ~ 93%. The sub-products are S-fluorinated derivatives of 2,5-diketopiperazine (6%) and tripeptide (1%). However, perfluoro-m-xylene is not a commercially available product. Its precursor is a perfluoro-m-xylene and perfluoro-o-xylene mixture, which reacts in the same way as HTL. Perfluoro-o-xylene stays intact in the reaction conditions. The fluorinated products of the reaction mixture were found to be poorly soluble in water at such high concentrations. It permitted us to precipitate them with water. The physicochemical characteristics of compounds are presented in the experimental part and supplementary materials.

The one-step synthetic pathway, using a system of DMSO, HTL hydrochloride, perfluoroarene, and triethylamine deprotection of the hydrochloride salt in various compound concentrations and conditions, is presented in Table 2. The simple reaction setup, use of fewer reactants, high yield, and fewer purification steps were considered essential factors. However, the reaction time is a major limitation of the one-step procedure. The increase in temperature allows the product to be obtained in ordinary time. Furthermore, a high base excess increases HTL self-condensation and N-substitution by triethylamine impurities, such as perfluoroarenes. In this way, we optimized the triethylamine excess and purified the base before synthesis (Table 2). Another possibility to reduce byproducts is using an expensive, sterically shielded N-ethylisopropylamine. 

**Figure 4:**
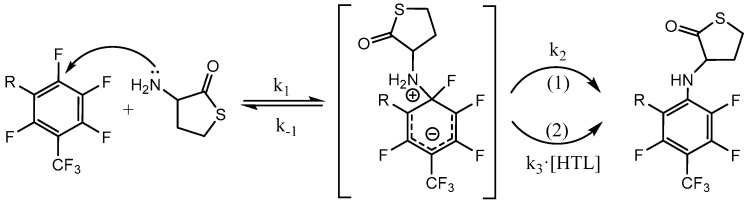
The possible mechanisms of reaction between PFT or PFX and HTL with (1) and without (2) basic catalysis by HTL excess. R = F or CF_3_.

**Figure 5:**
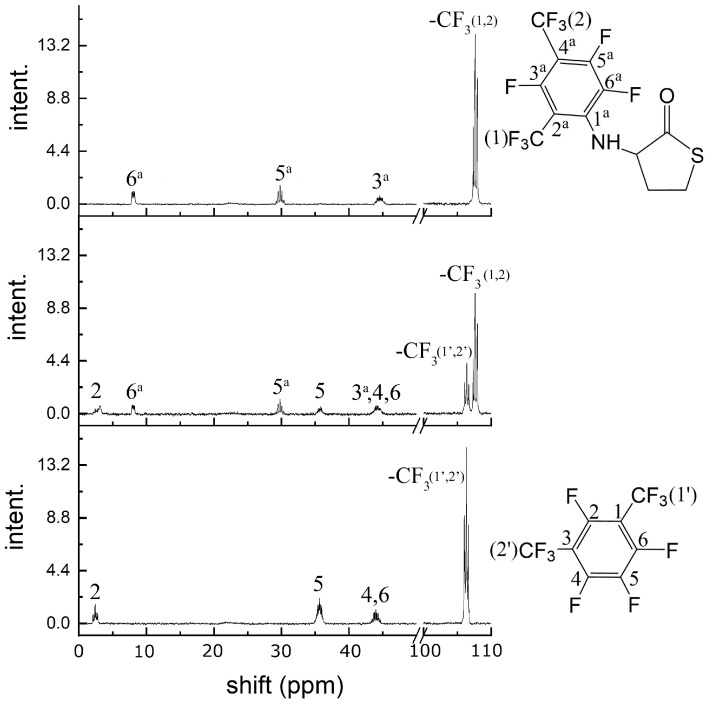
Typical ^19^F NMR spectra for PFX and HTL reaction in DMSO at 0 (**bottom**), 40 (**center**), and 163 (**top**) min. The reagent ratio PFX:HTL:NEt_3_ equals to 1:1.25:1.65.

**Figure 6:**
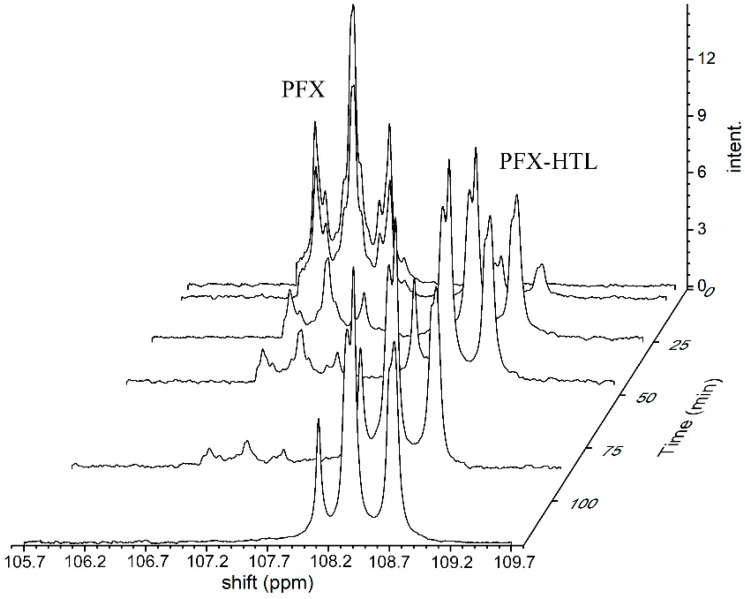
Typical ^19^F NMR spectra of the PFX and HTL reaction in DMSO in the presence of triethylamine in the CF_3_ groups’ shift diapason. The PFX:HTL:NEt3 ratio is 1:2.5:3.3.

To evaluate a reaction mechanism (Figure 4) between perfluoroarenes and HTL, various ratios of compounds were investigated by ^19^F NMR (Figure 5 and Figure 6). Higher concentrations (0.1 M) of perfluoroarenes were used for the reaction to proceed in a short time (Table S1). To identify the rate constant k_2_, the reactant steady state approximation was used. The kinetics curve in the perfluoroarene and HTL ratio~1:1 is well described by the mechanism one. The k_2_ value is 0.43 ± 0.02 M^−1^s^−1^ and 0.016 ± 0.001 M^−1^s^−1^ for PFX and PFT, respectively. However, for at least a 2-fold excess of HTL per PFX, the mechanism changes to number two (with HTL catalysis). 

### 2.2. Synthesis and Characterization of Fluorinated HSA Conjugates

**Figure 7:**
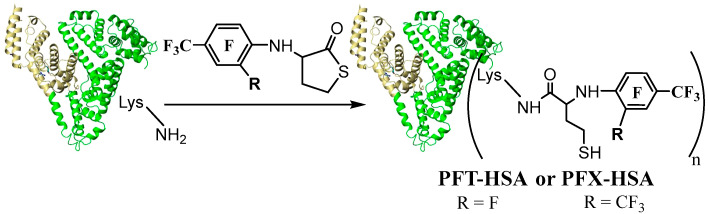
Synthetic route to PFT-HSA (*n* = 3.9 ± 0.1) and PFX-HSA (*n* = 4.9 ± 0.1) conjugates. Framework (shown schematically as a heart-like structure)—human serum albumin (HSA).

There are only a few works that handle covalent modification of HSA by fluorine-containing compounds for ^19^F MRI [39,40,58,64,82]. Yu et al. [40] and Zhu et al. [39] have selectively modified albumin on Cys34 residue. However, line broadening and signal splitting occurred during the work, hindering the ^19^F MRI application [40]. Recently [39], fluorinated tags with six CF_3_ groups (two perfluoro-tert-butyl moieties) were conjugated to Cys34, generating a low width ^19^F peak. However, only one molecule is possible to conjugate with albumin by such a procedure. In the present work, we present the multiple lysine labeling of albumin. N-fluorinated HTL was used as a site-specific acylation reagent. Previously, we used PFT HTL to synthesize albumin with 3.1 ± 0.1 fluorinated tags [64]. N-Homocysteinylation of HSA by PFT-HTL and PFX-HTL was performed under physiological-like conditions (PBS, 37 °C, pH 7.4) (Figure 7). Unreacted low-MW compounds were removed using Centricon concentrators by standard procedure. The yields of fluorinated HSA conjugates were 90–95%. For comparison, N-homocysteinylated HSA was synthesized. The mixture of S-substituted derivatives of PFT and PFT-HTL reacts with albumin in the same manner as pure PFT-HTL. S-substituted derivatives of PFT precipitate in water solutions and do not influence the reaction.

**Figure 8:**
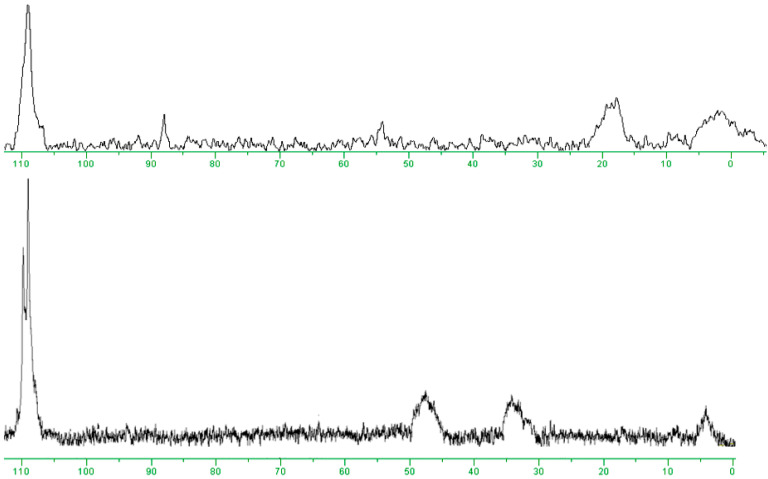
^19^F NMR spectra (300 MHz) of PFT-HSA (**top**) and PFX-HSA (**bottom**) conjugates in PBS at 37 °C.

The protein modification was proven by MALDI-ToF MS, UV spectroscopy, and ^19^F NMR. PFT-HSA and PFX-HSA contain 3.9 ± 0.1 and 4.9 ± 0.1 fluorinated residues, respectively. The ^19^F NMR spectra of PFT-HSA and PFX-HSA are presented in Figure 8. Multiple albumin labeling results in broad ^19^F signals. In the case of PFX-HSA, two CF_3_ groups of perfluoroxylene rings showed one split, but showed much more intense ^19^F signals than the PFT-HSA conjugate.

Protein over-labeling usually leads to significant changes in conformation, protein damage, loss of function, and precipitation. Wrong albumin modification may lead to protein oligomer formation and amyloidal transformation [65]. Therefore, we precisely controlled the protein labeling. Fluorinated albumin conjugates were characterized by gel electrophoresis (SDS-PAGE), circular dichroism (CD), and dynamic light scattering (DLS) (Table 3).

HSA solutions always contain some dimers, oligomers, and noncovalent, reversible dimer fractions (SDS PAGE Figure S2, Table 3) [83]. However, the amount of oligomer fraction is extremely high in the Hcy-HSA sample. N-homocysteinylation of HSA by natural HTL causes protein conformational changes and oligomer formation (Table 3, Hcy-HSA sample) [65,84]. On the contrary, the reaction with PFT-HTL and PFX-HTL leads only to slight changes in *α*-helical and *β*-sheet content and inhibits aggregation (Table 3, PFT-HSA and PFX-HSA samples). Despite the higher modification degree, the PFX-HSA conjugate better reproduces the properties of the native protein (Table 3, cf. HSA and PFX-HSA). The prevalent protein fraction is a monomer (~6 nm) in PFT-HSA and PFX-HSA samples, which is shown by number and volume modes by DLS (Figures S3 and S4, Table 3). The presence in the samples of a small number of large-sized particles can be seen in intensity mode, which is in good correlation with the native HSA sample (Figures S5 and S6). 

### 2.3. Cytotoxicity of HSA Conjugates

**Figure 9:**
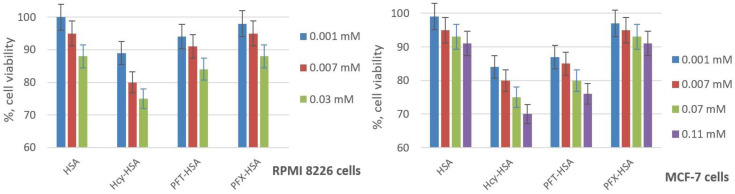
Effects of HSA conjugates on the viability of MCF-7 and RPMI 8226 cell lines. Cell viability was normalized using cells treated with PBS buffer as a 100% viability control. Hcy-HSA—*N*-homocysteinylated HSA containing 3 Hcy residues.

The ability of fluorinated HSA conjugates to cause cell damage was investigated by the widely-applied 3-(4,5-dimethylthiazol-2-yl)-2,5-diphenyltetrazolium bromide (MTT) test using breast cancer MCF-7 and human multiple myeloma RPMI 8226 cells [66,67]. During the exponential growth phase, cell cultures were treated for 72 h, with the amounts of the HSA conjugates in the range expected for MRI applications. The fluorinated HSA conjugates show no significant cell viability difference from native HSA (Figure 9), which indicates their high biocompatibility. On the contrary, the incubation of the cells with *N*-homocysteinylated HSA (Hcy-HSA) results in a reduction in cell viability [85]. The cell viability in RPMI cell lines is slightly higher for PFX-HSA than for PFT-HSA, and significantly higher for MCF-7 cells, which displays better possible prospects of perfluoroxylene-based albumin conjugation. 

### 2.4. Dual-Labeled Albumin Conjugates Synthesis

HSA has only one free SH group, Cys34, which can be used for site-specific modification [39,64]. However, the SH group of Cys34 may be in reduced form (in healthy patients, ~70%), oxidized as a disulfide (~25%), or a sulfinic or sulfonic acid (~5%). Ellman’s test indicated that the starting HSA in the present study contained 0.28 ± 0.05 sulfhydryl groups per protein molecule. Therefore, the albumin was treated with dithiothreitol (DTT) to convert the Cys-34 into free thiol used for labeling.

**Figure 10:**
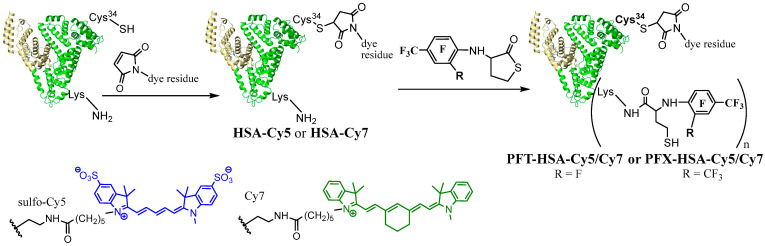
Synthetic route to PFT-HSA-Cy and PFX-HSA-Cy conjugates. Framework (shown schematically as a heart-like structure)—human serum albumin (HSA).

To synthesize dual-modified protein, we first obtained a fluorescently labeled fluorinated HSA (Figure 10). In order to visualize the cell uptake, HSA was labeled with Cy5 dye, which was shown to exhibit strong fluorescence for efficient identification of the molecule by flow cytometry [64]. The Cy7 is an optimal residue for further in vivo fluorescence imaging experiments [63]. Fluorescent dyes Cy7 or Cy5 were coupled to the single Cys-34 residue of albumin using the maleimide derivative of the dye via a Michael addition reaction. The HSA-Cy7 conjugate was modified by an *N*-homocysteinylation reaction using PFT-HTL or PFX-HTL compounds in PBS at 37 °C. PFT-HSA-Cy7 and PFX-HSA-Cy7 were characterized by different methods such as ^19^F NMR, MALDI ToF MS, UV, etc. (see experimental part). The cytotoxicity studies on RPMI and MCF-7 cell lines show the same good cell viability shown in Figure 9. The incubation of cells with PFT-HSA-Cy and PFX-HSA-Cy conjugates did not reveal any significant reduction in cell viability. The flow cytometry analysis shows almost the same cell internalization (~80%) for HSA-Cy5, PFT-HSA-Cy5, and PFX-HSA-Cy5 conjugates (Figure S7). The obtained data suggest that albumin modification does not influence cell penetration, and excellent cell viability studies are not related to low accumulation efficacy. Generally, our initial results have demonstrated the potential of PFT-HSA-Cy and PFX-HSA-Cy to serve as biocompatible optical and ^19^F MR imaging agents. ^19^F MRI is suitable for preoperative imaging studies. Fluorescence imaging is a powerful intraoperative tool for tumor margin demarcation [86,87]. Finally, it is important to incorporate multimodal imaging to precisely excise tumors.

### 2.5. Trypsin Degradation of Fluorinated HSA Conjugates

**Figure 11:**
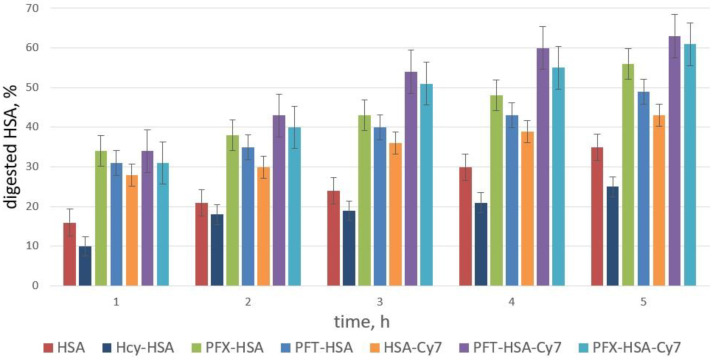
Susceptibility of HSA conjugates to tryptic proteolysis (enzyme/substrate ratio of 1:100). Samples were analyzed by SDS-PAGE. Albumin bands were visualized by Coomassie blue staining. Quantitative data were obtained by digitizing the gel (SDS-PAGE) using GelPro Analyzer software (Media Cybernetics). The bands in the gel lower than 66.5 kDa (HSA monomer MW) were marked as digested albumin (Figure S8).

A trypsin digest of the HSA and its conjugates was carried out under physiological-like conditions at pH 7.4 (PBS buffer). The reaction was monitored by SDS-PAGE. As shown in Figure 11, N-homocysteinylated HSA is degraded by trypsin slower than unmodified HSA, which is in good correlation with previously published data [88]. On the contrary, N-homocysteinylated albumin by PFT-HTL or PFX-HTL shows higher proteolysis susceptibility, which provides efficient biodegradation of fluorinated conjugates in the organism. PFT-HSA and PFX-HSA show the same tryptic digest efficiency. A similar pattern of susceptibilities to proteolysis was observed in PFT-HSA-Cy and PFX-HSA-Cy. As proteolysis proceeded, new peptide bands appeared with smaller molecular weights below HSA (Figure S8). HSA is degraded specifically into certain large fragments (~55 kDa) and much slower than the lower MW fragments ~45, 40, and 35 kDa. 

Proteases are enzymes that catalyze the hydrolysis of the peptide bonds in proteins. A number of the proteinases are secreted and active in the lysosomal compartment and extracellular matrix, where protease activity and concentration are highest. Many tumors have been shown to have elevated levels of proteases at an early stage [89]. Interestingly, persistent proteolysis has been observed in spreading tumors and metastasis [90]. The detection of a number of cancers may potentially benefit from protease imaging, including prostate, colon, brain, etc. [89,90,91,92]. Under the proteolysis condition in tumor tissue, heavy protein albumin (66.5 kDa) will decrease molecular weight, which highly influences the correlation time of fluorinated residues and ^19^F NMR peak width. Trypsinolysis generates a fast-motional regime for the fluorinated residues, and causes *T*_1_ and *T*_2_ relaxation time changes. The possible enzymatic hydrolysis of fluorinated albumin may serve as a stimuli-responsive ^19^F MRI probe [26]. However, such a proposition requires further research. An effective protease-activated switch system for nitroxide-labeled albumin and ^1^H MRI was evaluated [90,93]. Protein digestion switches from a stable, low-relaxivity state to one of high-relaxivity.

## 3. Materials and Methods

### 3.1. Chemicals

5,5′-dithio-bis(2-nitrobenzoic acid) (DTNB), DL-homocysteine thiolactone (HTL) hydrochloride, and all solvents and other reagents, unless stated differently, were purchased from Sigma (St. Louis, MO, USA) at the highest available grade and used without purification. MTT (3-[4,5-dimethylthiazol-2-yl]-2,5-diphenyltetrazolium bromide) assay kit was purchased from Invitrogen (Waltham, MA, USA). Centricon concentrators with a 3 kDa molecular weight cut-off were purchased from Millipore. Perfluoro-m-xylene (PFX) was prepared and kindly provided by V.E. Platonov (Novosibirsk Institute of Organic Chemistry, SB RAS). Cyanine dye derivatives were obtained from Lumiprobe (Moscow, Russia). Sequencing-grade modified trypsin for MALDI ToF peptide analysis was purchased from Promega (Madison, WI, USA) (cat. no. V5111). Trypsin for protein trypsinolysis was obtained from Gibco (Carlsbad, CA, USA) (cat. no. 15090046). The human serum albumin (HSA) used in this study was purchased from Sigma–Aldrich (St. Louis, MO, USA) (cat. no. A3782). MS (MALDI ToF) *m*/*z* HSA 66.48 kDa. Ellman’s test (SH group content) indicated that HSA in this study contained 0.28 ± 0.05 sulfhydryl groups per protein molecule.

### 3.2. Physicochemical Characterization

The number of thiol groups per albumin molecule was determined using the Ellman’s method, as described in the literature [94], or on the Thermo Scientific website with 5,5′-dithio-bis(2-nitrobenzoic acid) (DTNB) at pH 8 at 412 nm (ε = 1.4 × 10^4^ M^−1^cm^−1^), using a UV-1800 spectrometer with unmodified HSA as a control. DTNB produces a measurable, yellow-colored product when it reacts with free SH groups. 

Electronic absorption spectra were recorded on a UV-1800 spectrometer (Shimadzu, Tokyo, Japan). The concentrations of HSA solutions were determined by absorption at 278 nm, pH 7.4 (PBS), using the molar extinction coefficient ε = 3.7 × 10^4^ M^−1^cm^−1^. The concentrations of HTL solutions were determined by absorption at 240 nm using the molar extinction coefficient ε (PBS, pH 7.4; or 0.25 M carbonate buffer, pH 10.5) = (5.0 ± 0.1) × 10^3^ M^−1^cm^−1^.

Column chromatography was performed on silica gel 60 (0.063–0.200 mm) using CHCl_3_/(CH_3_)_2_CO 1:2.5 (*V*:*V*) and CHCl_3_ for elution. 

SDS-PAGE. Human serum albumin conjugates were analyzed by sodium dodecyl sulfate-polyacrylamide gel electrophoresis using 7% PAAG under Laemmli conditions with subsequent Coomassie Brilliant Blue staining. Quantitative data were obtained by digitizing the gel using GelPro Analyzer software 3.0.

NMR spectra were recorded at 25 °C in 5 mm NMR sample tubes. ^1^H, ^13^C, and ^19^F NMR spectra were recorded on an AV-300 NMR spectrometer (Bruker, Rheinstetten, Germany) at 300.13, 75.43, and 282.37 MHz, respectively. ^19^F NMR spectra for kinetics studies were recorded on a Spinsolve 80 NMR spectrometer (Magritek, Aachen, Germany) at 80 MHz. The chemical shifts were expressed in parts per million, ppm (δ). All ^1^H chemical shifts were calculated relative to the residual ^1^H NMR signal of the deuterated NMR solvent. C_6_F_6_ (δ = 0.00 ppm) was used as the external reference for chemical shifts in the ^19^F NMR spectra. The spin-spin coupling constants (J) are reported in hertz (Hz), and spin multiples are given as s (singlet), d (doublet), t (triplet), q (quartet), and m (multiplet). Abbreviation br. means broad. The coupling constants (J) in the difficult case were calculated by simulation of the spin system using the Spinworks freeware software package 4.0.5.

Circular dichroism (CD) data were collected at 25 °C with a JASCO J-600 spectrophotometer with a time constant of 4 s and a bandwidth of 1 nm, using a 0.01 cm path length quartz cell. All CD spectra were obtained from 190 to 240 nm, and the final spectrum was obtained as an average of 20 spectra. To determine the percentages of α-helices, β-sheets, and the disordered structures, we minimized the difference between the theoretical and experimental curves. The theoretical curves were calculated as a linear combination of the basis spectra of various components of the secondary structures taken from the CCA+ software [95]. For recording the CD spectra, samples consisted of 5 µM albumin in PBS. Changes in the secondary structure of the protein were examined by deconvolution of CD spectra to determine the α-helix and β-sheet content.

ESI MS was performed on a mass spectrometer, the LC/MSD Trap XCT (Agilent, Santa Clara, CA, USA).

MALDI-ToF mass spectra of proteins were recorded on a Bruker Autoflex Speed (Bruker, Germany) MALDI-TOF mass spectrometer in a positive linear mode. A smartbeam-II laser was used with 2,5-dihydroxyacetophenone (2,5-DHAP) as the matrix. Protein samples were desalted by ZipTip C4 pipette tips. A 2 µL protein sample solution was mixed with 2 µL of 2% TFA (trifluoroacetic acid). For the latter solution, 2 µL of the matrix (2,5-DHAP) was added. The mixture was pipetted up and down until the crystallization started. Approximately 1 μL of the resulting solution was deposited on the 384-grit steel target plate and allowed to dry before being introduced into the mass spectrometer. Mass spectra were obtained by averaging 2500–3000 laser shots. External calibration was provided by the [M+H]^+^ peak of human serum albumin at *m*/*z* 66,467. MALDI ToF data were deconvoluted using the software package mMass 5.5.0 [96,97,98]. 

Dynamic light scattering (DLS) measurements were carried out on a Malvern Zetasizer Nano device (Malvern Instruments, Worcestershire, UK) at 25 °C. The protein samples (50 µM) were prepared in PBS buffer, and measurements of their size were conducted.

### 3.3. Synthesis of S- and N-Fluorinated Derivatives of Homocysteine Thiolactone

#### 3.3.1. Synthesis of Homocysteine Thiolactone (HTL) Free Base

DL-homocysteine thiolactone hydrochloride (20.0 mg, 0.130 mmol) was stirred with 0.5 mL of preliminarily cooled 4 °C 0.25 M sodium carbonate buffer (pH 10.5) for 30 s. The alkali solution of HTL was quickly shaken with preliminary cooled to 4 °C ethyl acetate (13 mL), followed by organic solvent separation into a flask. The solvent was immediately removed in vacuo for 5–7 min. Evaporation was performed without a water bath. Under such conditions, the organic solvent HTL solution is self-cooled. After stopping the evaporation due to excessive cooling, the flask with the solution is lowered into a water bath (25 °C) until fogging disappears, and the procedure is repeated. The product was directly used for the next step owing to its instability even at temperatures below zero. The extraction efficiency was estimated by measuring the electronic absorption of the diluted water solutions of the given HTL solution before and after extraction (λ_max_ 240 nm; ε = (5.0 ± 0.1) × 10^3^ M^−1^cm^−1^). The efficiency of extraction was 87%. The thiolactone cycle retention rate during the extraction stage was 98%. The thiolactone cycle retention rate after extraction and evaporation was ~96%. The remaining 4% of compounds are 2,5-diketopiperazine of homocysteine (3%) and homocysteine tripeptide (1%).

#### 3.3.2. Reaction of HTL Free Base with Perfluorotoluene (PFT) and Perfluoro-m-xylene (PFX) (The Method № 1)

The synthetic procedure for PFT-HTL and PFX-HTL was adapted from Chubarov et al. [58,64]. For this synthesis, a free base of HTL obtained by a procedure in Section 3.3.1 was used. HTL (15.21 mg, 0.13 mmol) was quickly dissolved in 0.6 mL DMSO, and PFT (6.38 µL, 21.24 mg, 0.09 mmol) or 1,4- and 1,3-perfluoroxylene mixture (PFX) (11.22 µL, 0.12 mmol; 0.09 mmol of 1,3-PFX), or 1,3-PFX (8.1 µL, 0.09 mmol) was added. The mixtures were allowed to react at 25 °C for 14 h. After the reaction was completed, 0.4 mL of water was added to the solution, yielding a white precipitate. The mixture was centrifuged for 2 min at 6000× *g*, and the supernatant was discarded. The washing procedure was repeated at least one more time. The target compounds were separated by flash chromatography on silica gel 60 (0.063–0.200 mm) using CHCl_3_/(CH_3_)_2_CO 1:2.5 (*V*:*V*) and CHCl_3_ for elution. The PFX-HTL compound was not required for flash chromatography.

#### 3.3.3. Reaction of HTL Hydrochloride with Perfluorotoluene (PFT) and Perfluoro-m-xylene (PFX) in the Presence of a Triethylamine (The Method № 2)

The PFT (4.25 µL, 14.1 mg, 0.060 mmol) or 1,4- and 1,3-perfluoroxylene mixture (PFX) (8.09 µL, 0.09 mmol; 0.065 mmol of 1,3-PFX) and 13.5 µL of fresh anhydrous trimethylamine (refluxed under p-toluenesulfonyl chloride) were added to DL-HTL hydrochloride (10.00 mg, 0.065 mmol) dissolved in 6.5 mL of DMSO. The mixtures were allowed to react at 75 °C for 70 h. After cooling to ambient temperature, the reaction mixture was evaporated under reduced pressure using a liquid-nitrogen-cooled trap. After that, 0.4 mL of water was added to the solutions. The mixture was centrifuged for 2 min at 6000× *g*, and the supernatant was discarded. The procedure was repeated at least one more time. The target compound PFT-HTL was separated by flash chromatography on silica gel 60 (0.063–0.200 mm) using CHCl_3_/(CH_3_)_2_CO 1:2.5 (*V*:*V*) and CHCl_3_ for elution. The PFX-HTL compound was not required for flash chromatography. Perfluoro-o-xylene stays intact in the reaction conditions.

#### 3.3.4. N-(2,3,5,6-tetrafluoro-4-(trifluoromethyl)phenyl) Homocysteine Thiolactone (PFT-HTL)

UV-vis (CH_3_CN): λ_max_ 240 nm, ε = (1.3 ± 0.1) × 10^4^ M^−1^cm^−1^; 256 nm, ε = (2.1 ± 0.1) × 10^4^ M^−1^cm^−1^. ^19^F NMR (DMSO-d6): δ 108.91 (t, 3F, CF_3_), 17.46 (m, 2F, F-3a, F-5a), 5.02 (m, 2F, F-2a, F-6a), J_CF3,3a_ = J_CF3,5a_ = 20.2, J_2a,3a_ = J_5a,6a_ = 19.0, J_3a,6a_ = J_2a,5a_ = 5.0, J_NH,2a_ = J_NH,6a_ = 2.5, coupling constants (J) were calculated by simulation of the spin system using SpinWorks freeware software package. ^1^H NMR (DMSO-d6): δ 6.97 (br.d, 1H, NH), 4.75 (m, 1H, H-3), 3.42 (m, 1H, H-5′), 3.30 (m, 1H, H-5), 2.57 (m, 1H, H-4′), 2.33 (m, 1H, H-4), J_3,4_ = 12.8, J_3,4′_ = 7.0, J_3,5_ = 0.5, J_3,NH_ = 8.8, J_4,4′_ = 12.2, J_4,5_ = 7.0, J_4,5′_ = 12.2, J_4′,5_ = 1.3, J_4′,5′_ = 5.5, J_5,5′_ = 11.3, J_NH,2a_ = J_NH,6a_ = 2.5, J_4,NH_ = 2.0, J_4′,NH_ = 1.0. ^13^C NMR (CD_3_CN): δ 204.6 (C-2), 146.4 (C-2a,6a), 144.0 (C-3a,5a), 123.4 (C-1a), 109.0 (CF_3_), 108.5 (C-4a), 64.3 (C-3), 30.3 (C-4,4′ or 5,5′), 28.0 (C-5,5′ or 4,4′). ESI MS (m/z): calc. for C_11_H_5_NOSF_7_ [M-H]: 331.998, found 331.997. The yield of PFT-HTL was 57 % (method № 1), and 94 % (method № 2).

#### 3.3.5. N-(2,3,5-Trifluoro-4,6-bis(trifluoromethyl)phenyl) Homocysteine Thiolactone (PFX-HTL)

UV–vis (CH_3_CN): λ_max_ 255 nm ε = (1.3 ± 0.1) × 10^4^ M^−1^cm^−1^; 296 nm ε = (6.0 ± 0.1) × 10^3^ M^−1^cm^−1^; 325 nm ε = (1.9 ± 0.1) × 10^3^ M^−1^cm^−1^. ^19^F NMR (CD_3_CN): 108.78 (d, 3F, CF_3_(1)), 108.17 (t, 3F, CF_3_(2)), 45.18 (m, 1F, F-3a), 31.60 (m, 1F, F-5a), 7.91 (m, 1F, F-6a), J_CF3(1),3a_ = 30.9, J_CF3(1),5a_ = 3.6, J_CF3(2),3a_ = J_CF3(2),5a_ = 22.3, J_3a,5a_ = 2.6, J_3a,6a_ = 10.0, J_5a,6a_ = 18.0, J_NH,6a_ = 2.0. ^1^H NMR (DMSO-d6): 6.52 (br. d, 1H, NH), 4.71 (m, 1H, H-3), 3.43 (m, 1H, H-5′), 3.31 (m, 1H, H-5), 2.76 (m, 1H, H-4′), 2.26 (m, 1H, H-4), J_3,4_ = 12.8, J_3,4′_ = 7.0, J_3,5_ = 0.5, J_3,NH_ = 8.4, J_4,4′_ = 12.2, J_4,5_ = 7.0, J_4,5′_ = 12.2, J_4′,5_ = 1.3, J_4′,5 ’_= 5.3, J_5,5 ’_= 11.5, J_4,NH_ = 2.0, J_4′,NH_ = 1.0. ^13^C NMR (CD_3_CN): 207.2 (CO), 155.0 (C-3a), 141.6 (C-5a), 140.7 (C-6a), 138.3 (C-1a), 125.8 (CF_3_), 124.4 (CF_3_), 123.1 (C-4a), 121.6 (C-2a), 66.2 (C-3), 33.4 (C-5,5′), 28.0 (C-4,4′). ESI MS negative mode (m/z) calculated for C_12_H_5_NOSF_9_ [M-H]^-^: 381.995, found 381.896. The yield of PFX-HTL was 93 % (method № 1) and 99 % (method № 2).

### 3.4. Synthesis of N-homocysteinylated Albumin (Hcy-HSA)

The synthetic procedure for Hcy-HSA was adapted from Chubarov et al. [16]. Briefly, to 0.5 mL of a 0.53 mM solution of HSA in PBS (15 mM KH_2_PO_4_, 145 mM NaCl, pH 7.4 with 0.2 mM EDTA), a solution of a 6-fold molar excess of D,L-homocysteine thiolactone hydrochloride dissolved in 0.04 mL PBS. The reaction mixture was incubated at 37°C for 18 h with stirring at 500 rpm. The excess reagents were removed by filtration using Centricon concentrators (3 kDa molecular weight cutoff; Amicon Centriprep YM30, Millipore) according to the procedure described by the manufacturer, with PBS as the eluent. The yield of Hcy-HSA was ~90%. Hcy-HSA UV–vis (PBS, pH 7.4): λ_max_ 278. MALDI TOF MS m/z 66.86 kDa (~3.2 ± 0.1 Hcy residues). The number of SH groups per protein molecule was analyzed by Ellman’s assay, which is 3.37 ± 0.05 (excluding albumin-free SH group 3.09 ± 0.05).

### 3.5. Synthesis of HSA-Cy5 and HSA-Cy7

Commercially available HSA contains 0.1–0.7 free SH groups per protein, depending on the preparation method and plasma grade. According to our Ellmans’ test results, albumin A3782 contains 0.28 SH groups per protein molecule (see Materials subsection). To enlarge albumin modification by fluorescence dye, the reduction reaction by dithiothreitol (DTT) was performed [99]. Under this condition, no intramolecular disulfides (protein S-S bridges) were broken [99]. Using the Ellmans’ test, it was found that reduced albumin (HSA-SH) contains 0.9 ± 0.1 sulfhydryl group per protein molecule.

The synthetic procedure was adapted from Chubarov et al. [64]. Briefly, to 1 mL of a 1.0 mM solution of HSA-SH in PBS (15 mM KH_2_PO_4_, 145 mM NaCl, pH 7.4), add a solution of a 3-fold molar excess of sulfo-Cy5 maleimide or a 1.2-fold molar excess of a Cy7 maleimide derivative dissolved in 0.1 mL DMSO. During this process, the solution was stirred vigorously. The reaction mixture was incubated at 37 °C for 12 h with stirring at 500 rpm. The excess reagents were removed by filtration using Centricon concentrators (3 kDa molecular weight cutoff; Amicon Centriprep YM30, Millipore) according to the procedure described by the manufacturer, with PBS as the eluent. The yield of HSA-Cy5 and HSA-Cy7 was ~95%. HSA-Cy5 UV–vis (PBS, pH 7.4): λ_max_ 278, 652 nm (ε_652_ = (2.3 ± 0.1) × 10^5^ M^−1^cm^−1^, ~0.85 sulfo-Cy5 residues). HSA-Cy7 UV–vis (PBS, pH 7.4): λ_max_ 278, 762 nm (ε_762_ = (1.8 ± 0.1) × 10^5^ M^−1^cm^−1^, ~0.90 Cy7 residues).

### 3.6. Synthesis of Fluorinated Albumin Conjugates (PFT-HSA, PFX-HSA, PFT-HSA-Cy5, PFX-HSA-Cy5, PFT-HSA-Cy7, PFX-HSA-Cy7)

The synthetic procedure was adapted from Chubarov et al. [58,64]. Briefly, to 0.4 mL of the 1.15 mM solution of HSA or HSA-Cy in PBS (15 mM KH_2_PO_4_, pH 7.4, 145 mM NaCl), the solution of 20-fold molar excess of N-(2,3,5,6-tetrafluoro-4-(trifluoromethyl)phenyl) homocysteine thiolactone (PFT-HTL) or -(3,5,6-trifluoro-2,4-bis(trifluoromethyl)phenyl) homocysteine thiolactone (PFX-HTL) in 150 µL DMSO was added. The reaction mixture was incubated at 37 °C for 20 h with stirring at 300 rpm. The excess reagents were removed by filtration using Centricon concentrators (3 kDa molecular weight cutoff; Amicon Centriprep YM30, Millipore) according to the procedure described by the manufacturer, with PBS as the eluent.

PFT-HSA. The yield was ~90%. UV-vis (PBS, pH 7.4): λ_max_ 256 ε_256_ = (9.7 ± 0.1) × 10^4^ M^−1^cm^−1^. ^19^F NMR (PBS, pH 7.4): δ 110.7–106.1 (br. m., max 108.9 ppm, CF_3_), 10.0–20.0 (br. m., max 16.6 ppm, F-3, F-5), −5.0–5.0 (br. m., max 0.0 ppm, F-2, F-6). MALDI TOF MS *m*/*z* 67.8 kDa (~3.95 ± 0.05 PFT-Hcy residues). The average amount of PFT-Hcy residue per protein molecule by UV and MALDI TOF MS is 3.9 ± 0.1.

PFT-HSA-Cy5. The yield was ~92%. UV-vis (PBS, pH 7.4): λ_max_ 256, 652 nm ε_652_ = (2.3 ± 0.1) × 10^5^ M^−1^cm^−1^. MALDI TOF MS *m*/*z* 68.5 kDa. 

PFT-HSA-Cy7. The yield was ~90%. UV-vis (PBS, pH 7.4): λ_max_ 256, 762 nm ε_762_ = (1.8 ± 0.1) × 10^5^ M^−1^cm^−1^. MALDI TOF MS *m*/*z* 68.4 kDa.

PFX-HSA. The yield was ~95%. UV-vis (PBS, pH 7.4): λ_max_ 256, 278, 300 ε_256_ = (9.7 ± 0.1) × 10^4^ M^−1^cm^−1^. ^19^F NMR (PBS, pH 7.4): δ 109.0 (br. m., CF_3_(1), CF_3_(2)), 47.0 (br. m., F-3), 33.8 (br. m., F-5), 4.0 (br. m., F-6). MALDI TOF MS *m*/*z* 68.4 kDa (~5.0 ± 0.1 PFX residues). The average amount of PFX-Hcy residue per protein molecule by UV and MALDI TOF MS is 4.9 ± 0.1.

PFX-HSA-Cy5. The yield was ~90%. UV-vis (PBS, pH 7.4): λ_max_ 256, 278, 300, 652 nm ε_652_ = (2.3 ± 0.1) × 10^5^ M^−1^cm^−1^. MALDI TOF MS *m*/*z* 69.1 kDa. 

PFX-HSA-Cy7. The yield was ~90%. UV-vis (PBS, pH 7.4): λ_max_ 256, 278, 300, 762 nm ε_762_ = (1.8 ± 0.1) × 10^5^ M^−1^cm^−1^. MALDI TOF MS *m*/*z* 69.0 kDa. 

### 3.7. Synthesis of PFT-HSA Using the Mixture of S- and N-Substituted Derivatives of PFT

To 0.4 mL of the 1.15 mM solution of HSA in PBS, the solution of a 20-fold molar excess of PFT-HTL with various amounts of S-substituted derivatives of PFT in 150 µL DMSO was added. The reaction mixture was incubated at 37 °C for 20 h with stirring at 300 rpm. The excess reagents were removed by filtration using Centricon concentrators (3 kDa molecular weight cutoff; Amicon Centriprep YM30, Millipore) according to the procedure described by the manufacturer, with PBS as the eluent.

### 3.8. Susceptibility of Albumin Conjugates to Proteolysis

The solution of HSA and HSA conjugates 50 µL of 1 mM were digested with 2.5 µL trypsin («Gibco» cat. no. 15090046) at an enzyme-substrate ratio of 1:100 in PBS buffer (pH 7.4) for 0.5–6 h at 37 °C. Digest aliquots of 3.75 µL were diluted with 71.25 µL of PBS. The latter solution aliquot (7.5 µL) was mixed 1:1 with SDS-PAGE sample buffer (50 mM tris hydrochloride pH 6.8, 1% SDS, 10% glycerol, and 0.025% bromophenol blue with or without 0.1% DTT), denatured for 5 min at 95 °C, and subjected to SDS-PAGE on 10% gels. All samples were analyzed by SDS-PAGE on the same day. Protein bands were visualized by staining with Coomassie Brilliant Blue. Quantitative data were obtained by digitizing the gel using GelPro Analyzer software.

### 3.9. Cell Culture and Toxicity Assay

Cell Culture. Tumor cell lines from human mammary adenocarcinoma MCF-7 and human glioblastoma T98G were cultured in IMDM and EMEM medium, respectively, supplemented with 10% fetal bovine serum (FBS) (Invitrogen), penicillin (100 units/mL), and streptomycin (100 μg/mL) at 37 °C with 5% CO_2_ in a humid atmosphere. 

The inhibition of cell proliferation was determined using a colorimetric assay based on the cleavage of MTT (3-(4,5-dimethylthiazol-2-yl)-2,5-diphenyltetrazolium bromide) by mitochondrial dehydrogenases in viable cells, leading to formazan formation [67]. Briefly, exponentially growing cells were plated in a 96-well plate (2000 cells per well). After overnight incubation, the cells were treated with media containing albumin conjugates. The solutions of conjugates were applied into the media for 72 h at 37 °C. A 10 μL aliquot of MTT solution (25 mg/mL in PBS) was added to each well, and the plates were incubated at 37 °C for 3 h. The medium was removed, and the dark blue crystals of formazan were dissolved in 0.1 mL of isopropanol. The absorbance at 570 nm (peak) and 620 nm (baseline) was read using a microplate reader, Multiscan EX (Thermo Electron Corporation). The results were expressed as a percentage of the control values. All values in the present study are given as the mean ± standard deviation (SD) values, and all measurements were repeated three times.

## 4. Conclusions

Human serum albumin (HSA) is an attractive protein for targeted delivery. Recently, various albumin-binding prodrugs and probes or covalent constructions have also been developed for cancer treatment [10,46,54,100]. The fluorinated HSA has great potential for ^19^F MRI diagnostics and therapy [39,58,63,64]. Herein, a new family of fluorinated albumin conjugates using perfluo-m-xylene moieties was synthesized. These conjugates retain important biocompatible properties of albumin, such as high cell viability and cell internalization. Sparing perfluo-m-xylene conjugation through HTL natural acylation reagent, leads to the preservation of HSA physical properties, such as 3D dimensional structure and oligomerization susceptibility. The combination of good biodegradation in cancer tissue and the long blood half-life of albumin can provide a possible enzyme-activatable ^19^F MRI probe, which warrants further investigation. In conclusion, the fluorinated albumin species have a high potential for ^19^F MRI tumor diagnostics. Nevertheless, further optimization of fluorine tags is required to prove a single low-width ^19^F peak. An extended biomedical study on in vivo models is indispensable for clinical potential examination.

## Figures and Tables

**Table 1:** A brief summary of HTL free base synthesis results.

Synthesis Step	Conditions		Compounds Yield, %		
	C_HTL_, M	Time, min	HTL	2,5-Diketopiperazine	Peptide
Extraction from aqueous solution (pH 10.5) ^a^	0.40 ^a^	0.5 ^a^	91	9	
	0.26	0.5	98	1–2	
	0.20	0.5	98	1–2	
	0.40	10	80	20	
	0.26	10	90	10	
	0.26	30	60	40 ^b^	
Evaporation in vacuo	C_HTL_ = 0.26 M,				
	self-cooling ^c^	0.5 min	96 ^d^	3	1
	water bath 25 °C	0.5 min	87	11	2
	water bath 40 °C	0.5 min	73	24	3
	C_HTL_ = 0.26 M, water bath 25 °C	10 min	81	15	5
	C_HTL_ = 0.40 M, water bath 25 °C	0.5 min	77	15	8
	C_HTL_ = 0.40 M, water bath 25 °C	10 min	65	20	15
Compound storage in solid state	without storage		96 ^d^	3	1
	−20 °C	1 day	94	5	1
	−20 °C	1 week	89	9	2
	4 °C	1 day	88	11	1
	25 °C	1 day	75	13	2

^a^ HTL extraction from 0.25 M sodium carbonate buffer (pH 10.5) using cooled ethyl acetate (4 °C). C_HTL_ and time are presented for aqueous buffer incubation. The HTL compound quantity was estimated by calculation of adsorption by UV spectroscopy of the water solution aliquots on the wavelength 240 nm (HTL, ε_240_ = (5.0 ± 0.1) × 10^3^ M^−1^cm^−1^); ^b^ White precipitate of homocysteine 2,5-diketopiperazine -S-S- polymer formation. The compound is not soluble in water, or ordinary solvents. The structure was defined using a DTT compound for S-S bond reduction with subsequent analysis by ^1^H NMR (DMSO-d6, 300 MHz); ^c^ Evaporation of ethyl acetate without a water bath. Under such conditions, the organic solvent HTL solution is self-cooled. After stopping the evaporation due to excessive cooling, the flask with the solution is lowered into a water bath (25 °C) until fogging disappears, and the procedure is repeated; ^d^ The ratio was calculated by ^1^H NMR (DMSO-d6, 300 MHz).

**Table 2:** The ingredients, reaction conditions, and products of the reaction between HTL and perfluoroarenes in the presence of triethylamine in DMSO.

Conditions	T, °C	Time, h	Compounds Ratio per Perfluoroarene by ^19^F NMR, %			
			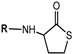	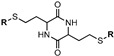	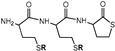	Initial PFT or PFX
PFT 0.01 M, HTL 0.02 M, NEt_3_ 0.04 M	25	20	35	0	0	65
		45	60	5	3	32
		120	83	11	6	0
PFT 0.02 M, HTL 0.02 M, NEt_3_ 0.04 M	25	20	31	0	0	69
		45	50	2.5	0.5	47
		70	61	3	1	35
		120	83	5.5	1.5	10
		150	92.5	6	1.5	0
PFT 0.01 M, HTL 0.02 M, NEt_3_ 0.08 M	25	20	30	0	0	70
		45	50	3.5	1.5	45
		150	87	8	5	0
PFT 0.02 M, HTL 0.02 M, NEt_3_ 0.04 M	50	20	72	2	1	25
		45	88	3	1.5	7.5
		70	90	6.5	3.5	0
PFT 0.009 M, HTL 0.01 M, NEt_3_ 0.013 M	75	20	63	1	0.5	35
		45	84	3	1	12
		70	94	5	1	0
PFX 0.009 M, HTL 0.01 M, NEt_3_ 0.013 M	75	15	~100	~0	0	0

**Table 3:** Fluorinated HSA characteristics.

HSA Type		CD Analysis		DLS Analysis	SDS-PAGE Analysis	
	*n*	α-Helix, %	β-Sheet, %	Hydrodynamic Diameter, nm	Oligomer, %	Monomer, %
HSA	-	60.0	4.5	6.1 ± 0.6 ^1^	7.0	93.0
PFT-HSA	3.9 ± 0.1	56.4	6.8	6.2 ± 0.8	8.7	91.3
PFX-HSA	4.9 ± 0.1	58.2	5.0	6.0 ± 0.9	7.5	92.5
Hcy-HSA	2.9 ± 0.1	50.0	10.0	n.d.	83.0	17.0

^1^ DLS data of 50 µM HSA conjugates in PBS by Number mode.

## Data Availability

Not applicable.

## Supplementary Materials

The following supporting information can be downloaded at: https://www.mdpi.com/article/10.3390/molecules28041695/s1, Figure S1: Structures of nonfluorinated and S-fluorinated 2,5-diketopiperazine and tripeptide derivatives; Table S1: The ingredients and reaction conditions of the reaction between HTL and perfluoroarenes for mechanism studies; Figure S2: SDS–PAGE of PFT-HSA (line 1), PFX-HSA (line 2), HSA (line 3), and Hcy-HSA (line 4) conjugates; Figure S3: DLS size distribution of A—PFT-HSA; B—PFX-HSA by number; Figure S4: DLS size distribution of A—PFT-HSA; B—PFX-HSA by volume; Figure S5: DLS size distribution of A—PFT-HSA; B—PFX-HSA by intensity; Figure S6: DLS size distribution of HSA by intensity; Figure S7: Cellular uptake of fluorescently labeled albumin conjugates; Figure S8: Typical albumin proteolysis SDS-PAGE data.

## Sample Availability

Samples of the compounds are available from authors.
