# Supplementary material for: Fluorinated Human Serum Albumin as Potential 19F Magnetic Resonance Imaging Probe
Source: Molecules. 2023 Feb 10;28(4):1695. doi: 10.3390/molecules28041695 (PMC9959765; doi:10.3390/molecules28041695)
Supplement: Supplementary file 1 [file molecules-28-01695-s001.zip › molecules-2209261-supplementary.pdf]

Supplementary Materials

Fluorinated Human Serum Albumin as Potential ^{19}F Magnetic Resonance Imaging Probe

Dmitry E. Mitin [1,2], and Alexey S. Chubarov [1,2,*]

[1] Institute of Chemical Biology and Fundamental Medicine SB RAS, 630090 Novosibirsk, Russia;

[2] Novosibirsk State University, 630090 Novosibirsk, Russia.

Figure S1. Structures of nonfluorinated and S-fluorinated 2,5-diketopiperazine and tripeptide derivatives. 2

Table S1. The ingredients and reaction conditions of the reaction between HTL and perfluoroarenes for mechanism studies..4

HTL pKa determinations 6

Figure S2. SDS–PAGE of PFT-HSA (line 1), PFX-HSA (line 2), HSA (line 3), and Hcy-HSA (line 4) conjugates under Laemmli condition without addition of DTT with subsequent Coomassie blue staining. Bands corresponding to monomeric protein (MW = 66.5 kDa) and its dimer (MW ~ 130 kDa) were observed in samples. 6

Figure S3. DLS size distribution of A – PFT-HSA; B – PFX-HSA by Number. 6

Figure S4. DLS size distribution of A – PFT-HSA; B – PFX-HSA by Volume...7

Figure S5. DLS size distribution of A – PFT-HSA; B – PFX-HSA by Intensity. 7

Figure S6. DLS size distribution of HSA by Intensity. 7

Figure S7. Cellular uptake of fluorescently labeled albumin conjugates. 7

Figure S8. Typical albumin proteolysis SDS-PAGE data. Susceptibility of HSA and its conjugates to tryptic proteolysis (enzyme/substrate ratio 1:100). The bands in the gel lower 66.5 kDa (HSA MW) were marked as digested albumin. Quantitative data were obtained by digitizing the gel (SDS-PAGE) using GelPro Analyzer software (Media Cybernetics). SDS-PAGE (10%), pt. 6 h. The gel was stained with Coomassie Brilliant Blue. Line 1, MW markers (Sigma S8445); line 2, PFX-HSA; line 3, PFT-HSA; line 4, HSA; line 5, Hcy-HSA. 8

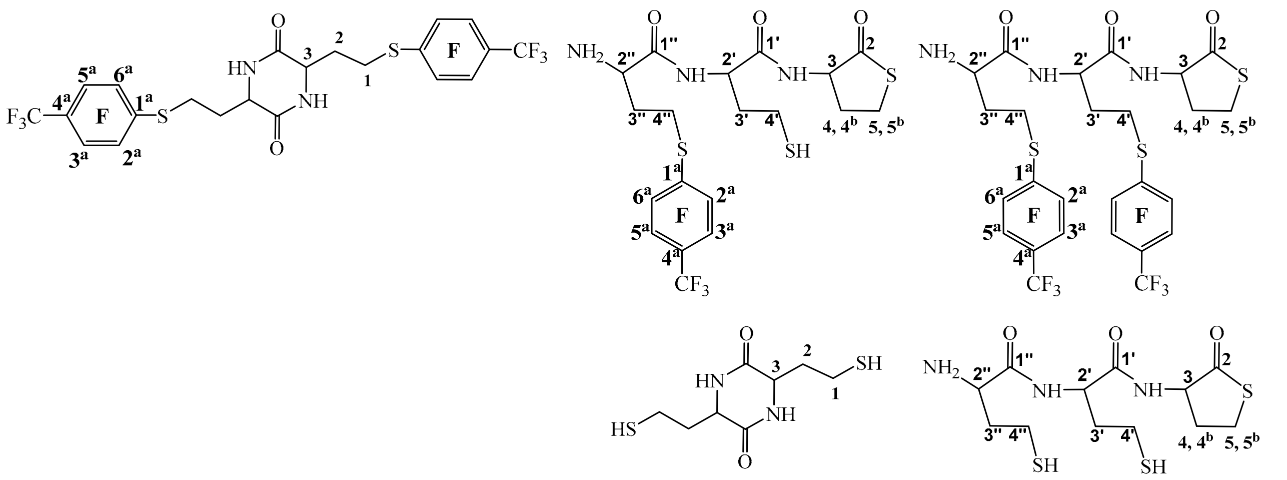

Figure S1. Structures of nonfluorinated and S-fluorinated 2,5-diketopiperazine and tripeptide derivatives.

3,6-Bis(2-(2,3,5,6-tetrafluoro-(trifluoromethyl)phenylthio)ethyl) piperazine-2,5-dione

UV-vis (CH_3CN): λ_{max} 279 nm (ε = (1.9 ± 0.1) × 10^3 M^{-1} cm^{-1}). ^{19}F NMR (CD_3CN): δ 107.12 (t, 6F, CF_3), 30.12 (m, 4F, F-2^a, F-6^a), 20.90 (m, 4F, F-3^a, F-5^a), $J_{CF3,\ 3a\ and\ 5a}$ =21.4. ^{1}H NMR (CD_3CN): δ 6.68 (br.s, 2H, NH), 3.95 (t, 2H, H-3), 3.10 (m, 4H, H-1), 1.92 (m, 4H, H-2), $J_{1,2}$=7.5, $J_{2,3}$=6.0. ^{13}C NMR (CD_3CN): δ 168.6 (CO), 149.4 (C-2^a, C-6^a), 147.0 (C-3^a, C-5^a), 120.7 (C-1^a), 113.2 (C-4^a), 109.0 (CF_3), 54.2 (C-3), 34.8 (C-2), 30.5 (C-1). ESI MS negative mode (m/z) calculated for $C_{22}H_{12}N_2O_2S_2F_{14}$ [M]: 666.012, found 665.891; [M+K-2H]: 702.960, found 702.891.

***S*-(*p*-perfluorotolyl)-homocysteinyl-*S*-(*p*-perfluorotolyl) homocysteinyl-homocysteine thiolactone**

UV-vis (CH_3CN): λ_{max} 240 nm, 279 nm.^{19}F NMR (DMSO-d_6): δ 107.08 (t, $J_{CF3,3a\ and\ 5a}$ = 21.8, 6F, CF_3), 30.30 (m, 4F, F-2^a, F-6^a), 20.97 (m, 4F, F-3^a, F-5^a). ^{1}H NMR (DMSO-d_6): δ 4.54 (dd, 1H, J_4= 12.8, J_{4b}=7.0, H-3), 4.40 (m, 1H, H-2'), 3.34 (m, 1H, H-2''), 3.33 (m, 1H, H-5), 3.25 (m, 1H, H-5^b), 3.21 (m, 4H, H-4', H-4''), 2.37 (m, 1H, H-4^b), 2.05 (m, 4H, H-3', H-3''), 1.85 (m, 1H, H-4). ^{13}C NMR (CD_3CN): δ 204.6 (C-2), 178.2 (C-1', C-1''), 149.3 (C-2^a, C-6^a), 146.9 (C-3^a, C-5^a), 120.7 (C-1^a), 113.2 (C-4^a), 109.0 (CF_3), 64.2 (C-3), 54.4 (C-2'), 54.0 (C-2''), 33.9 (C-3', C-3''), 32.3 (C-4', C-4''), 30.3 (C-5), 27.6 (C-4). ESI MS positive mode (*m/z*) calculated for $C_{26}H_{20}N_3O_3S_3F_{14}$ [M+H]: 784.044, found 783.790; [M+Na]: 806.026, found 805.789.

***S*-(p-perfluorotolyl)homocysteinyl-homocysteinyl-homocysteine thiolactone**

UV-vis (CH_3CN): λ_{max} 240 nm, 279 nm. ^{19}F NMR (DMSO-d_6) δ: 107.10 (t, $J_{CF3,3c}$=21.2, 3F, CF_3), 30.31 (m, 2F, F-2^a, F-6^a), 20.98 (m, 2F, F-3^a, F-5^a). ^{1}H NMR (DMSO-d_6) δ: 4.48 (dd, 1H, J_4= 12.8, J_{4b}=7.0, H-3), 4.34 (m, 1H, H-2'), 3.34 (m, 1H, H-2''), 3.28 (m, 1H, H-5), 3.25 (m, 1H, H-5^b), 3.21 (m, 2H, H-4''), 2.50 (m, 2H, H-4'), 2.37 (m, 1H, H-4^b), 2.05 (m, 2H, H-3''), 1.87 (m, 2H, H-3'), 1.83 (m, 1H, H-4). ^{13}C NMR (CD_3CN) δ: 204.6 (C-2), 178.2 (C-1', C-1''), 149.3 (C-2^a, C-6^a), 146.9 (C-3^a, C-5^a), 120.7 (C-1^a), 113.2 (C-4^a), 109.0 (CF_3), 64.2 (C-3), 54.2 (C-2'), 54.0 (C-2''), 33.9 (C-3''), 32.3 (C-4''), 31.0 (C-3'), 30.3 (C-5), 27.6 (C-4),

21.0 (C-4’). ESI MS negative mode (m/z) calculated for $C_{19}H_{19}N_3O_3S_3F_7$ [M-H]: 566.048, found 565.893; [M+Na-H]: 589.037, found 588.887.

Homocysteine 2,5-diketopiperazine

^{1}H NMR (DMSO-d6): δ 8.20 (br. s, 2H, NH), 3.90 (t, 2H, H-3), 2.40 (t, 2H, H-1), 1.95 (m, 4H, H-2), $J_{1,2}$=7.5, $J_{2,3}$=6.1. ^{13}C NMR (DMSO-d6): δ 168.5 (CO), 53.0 (C-3), 36.4 (C-2), 19.0 (C-1). ESI MS (m/z) calculated for $C_8H_{13}N_2O_2S_2$ [M-H]: 233.042, found 233.041.

Homocysteinyl homocysteinyl-homocysteine thiolactone

^{1}H NMR (DMSO-d_6): δ 4.50 (dd, 1H, J_4= 12.7, J_{4b}=7.1, H-3), 4.40 (m, 1H, H-2’), 3.33 (m, 1H, H-2’’), 3.30 (m, 1H, H-5), 3.25 (m, 1H, H-5^b), 2.40 (m, 4H, H-4’, H-4’’), 2.36 (m, 1H, H-4^b), 1.95 (m, 4H, H-3’, H-3’’), 1.85 (m, 1H, H-4). ^{13}C NMR (CD_3CN): δ 203.0 (C-2), 178.0 (C-1’, C-1’’), 64.6 (C-3), 55.4 (C-2’), 54.0 (C-2’’), 33.8 (C-3’, C-3’’), 30.3 (C-5), 27.6 (C-4), 20.0 (C-4’, C-4’’).

Table S1. The ingredients and reaction conditions of the reaction between HTL and perfluoroarenes for mechanism studies.

Entry	C(PFX), M	C(HTL), M	C(NEt_3), M	Ratio (PFX:HTL:NEt_3)	k_{eff}, $M^{-1}s^{-1}$
1	0.2	0.5	0.66	1:2.5:3.3	See below
2	0.1	0.125	0.165	1:1.25:1.65	0.43 ± 0.01
3	0.1	0.125	0.5	1:1.25:5	0.399 ± 0.005
4	0.1	0.04125	0.165	1:0.4125:1.65	0.46 ± 0.02
	C(PFT), M	C(HTL), M	C(NEt_3), M	Ratio (PFT:HTL:NEt_3)	
5	0.1	0.125	0.5	1:1.25:5	0.0151 ± 0.0002
6	0.1035	0.1	0.132	1.035:1:1.32	0.0160 ± 0.0004

Mechanism (1)

PFX experiments data | PFT experiments data

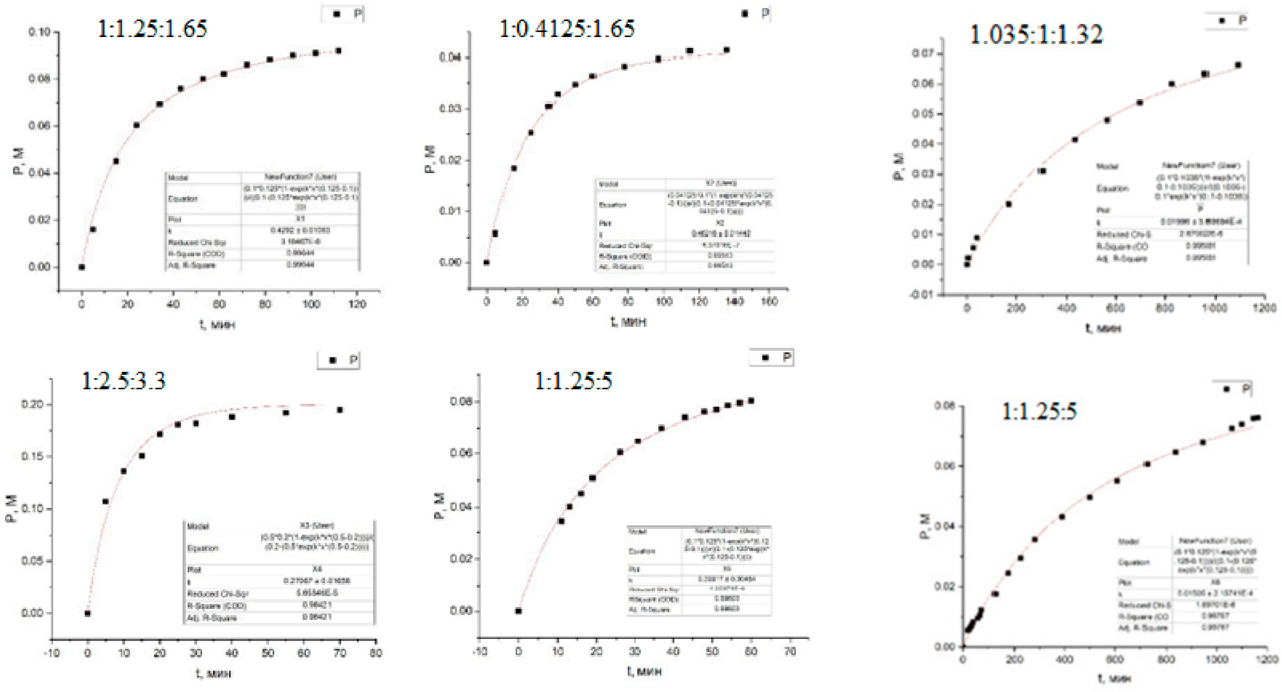

The experiment with PFX:HTL:NEt_3 concentration ratio of 1:2.5:3.3 is not realized by mechanism (1). Such reaction behavior may be calculated by mechanism (2) (Figure 4).

Mechanism (2) PFX:HTL:NEt_3 = 1:2.5:3.3

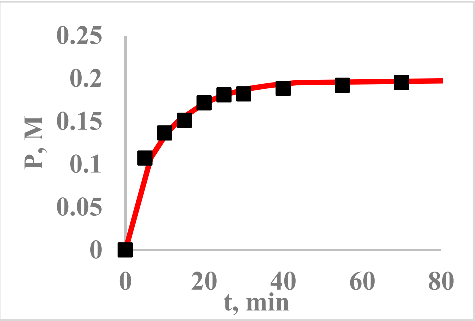

Mechanism (1) Figure 4. Derivation of the equation P(t) by mechanism (1):

$$\begin{cases} \frac{dA}{dt} = \frac{dB}{dt} = k_{-1}C - k_1AB \\ \frac{dC}{dt} = k_1AB - (k_{-1} + k_2)C \approx 0 \;\; => C = \frac{k_1}{k_{-1} + k_2}AB \\ \frac{dP}{dt} = k_2C \end{cases}$$

$$\frac{dA}{dt} = \left(k_{-1} - \frac{k_1}{k_{-1} + k_2}\right)AB = -k_{eff} \cdot AB \qquad (1)$$

$$\frac{dA}{dt} = -\frac{dP}{dt} = -k_{eff} \cdot (A_0 - P)(B_0 - P) \qquad (2)$$

$$\frac{1}{A_0 - B_0} \ln\left[\frac{B_0}{A_0}\frac{(A_0 - P)}{(B_0 - P)}\right] = k_{eff} \cdot t \qquad (3)$$

$$P(t) = \frac{A_0\left(exp\left[(A_0 - B_0)k_{eff} \cdot t\right] - 1\right)}{\frac{A_0}{B_0}\, exp\left[(A_0 - B_0)k_{eff} \cdot t\right] - 1} \qquad (4)$$

Mechanism 2 Figure 4. Derivation of the equation P(t) by mechanism (2):

$$\frac{dC}{dt} = k_1AB - (k_{-1} + k_2B)C \cong 0 \;\; => \;\; C = \frac{k_1}{k_{-1} + k_2B}AB \qquad (10)$$

$$\frac{dP}{dt} = k_2CB = \frac{k_1k_2}{k_{-1} + k_2B}AB^2 \qquad (11)$$

$$\left[\frac{k_{-1}}{(A_0 - P)(B_0 - P)^2} + \frac{k_2}{(A_0 - P)(B_0 - P)}\right]dP = k_1k_2 \cdot dt \qquad (12)$$

$$k_{-1}\int_0^{P(t)} \left(\frac{11{,}11}{0{,}2 - P} - \frac{11{,}11}{0{,}5 - P} - \frac{3{,}33}{(0{,}5 - P)^2}\right) + k_2 \cdot 3{,}33\left(\frac{1}{A_0 - P} - \frac{1}{B_0 - P}\right)dP = k_1k_2\int_0^t dt \qquad (13)$$

$$t = \left(\frac{11{,}11 \cdot k_{-1}}{k_1k_2} + \frac{3{,}33}{k_1}\right)\left[ln\frac{0{,}2}{0{,}2 - P(t)} - ln\frac{0{,}5}{0{,}5 - P(t)}\right] - \frac{3{,}33 \cdot k_{-1}}{k_1k_2}\left(\frac{1}{0{,}5 - P(t)} + 2\right) =$$

$$= a\left[ln\frac{0{,}2}{0{,}2 - P(t)} - ln\frac{0{,}5}{0{,}5 - P(t)}\right] - b\left(\frac{1}{0{,}5 - P(t)} + 2\right) \qquad (14)$$

The closest approximation occurs at a = 13,709 and b = 0,175.

$$\left.\begin{cases} a = 13{,}709 = \frac{11{,}11 \cdot k_{-1}}{k_1k_2} + \frac{3{,}33}{k_1} \\ b = 0{,}175 = \frac{3{,}33 \cdot k_{-1}}{k_1k_2} \end{cases}\right\} => 0{,}584 + \frac{3{,}33}{k_1} = 13{,}709 => k_1 = 0{,}2537\; M^{-1}s^{-1}$$

$$\frac{k_{-1}}{k_1 k_2} = \frac{k_{-1}}{0{,}2537 \cdot k_2} = \frac{0{,}175}{3{,}33} = 0{,}05255 => \frac{k_2}{k_{-1}} = 75$$

$k_1 = 0{,}2537$ $M^{-1}s^{-1}$ and $k_2/\ k_{-1} = 75$.

HTL pKa determinations

The pKa of HTL was determined at 25 °C, 30 °C, and 37 °C by potentiometric titration of the hydrochloride salt of HTL (1 mM) with NaOH (8 mM). For ionic strength adaptation, sodium chloride was used. pH was monitored by using a Sartorius PP-15 pH meter (Sartorius, Germany).

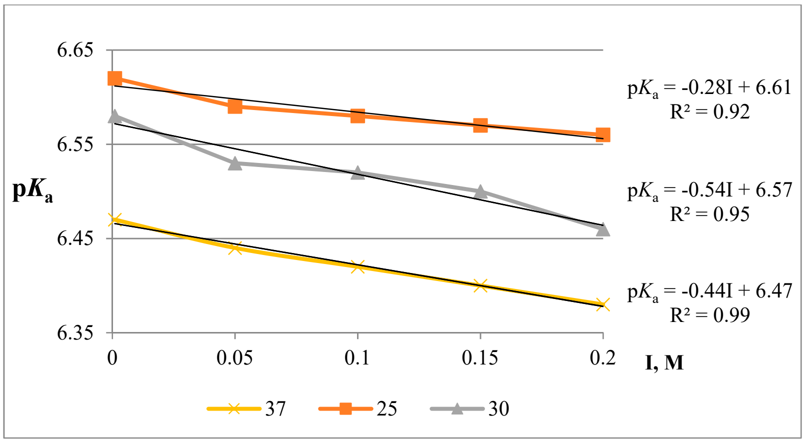

I = 0.167 M (for PBS buffer). pKa (25 °C) = 6.56, pKa (30 °C) = 6.48, pKa (37 °C) = 6.40,

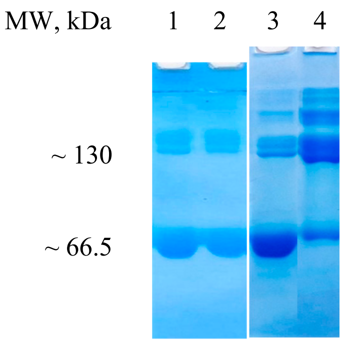

Figure S2. SDS–PAGE of PFT-HSA (line 1), PFX-HSA (line 2), HSA (line 3), and Hcy-HSA (line 4) conjugates under Laemmli condition without addition of DTT with subsequent Coomassie blue staining. Bands corresponding to monomeric protein (MW = 66.5 kDa) and its dimer (MW ~ 130 kDa) were observed in samples.

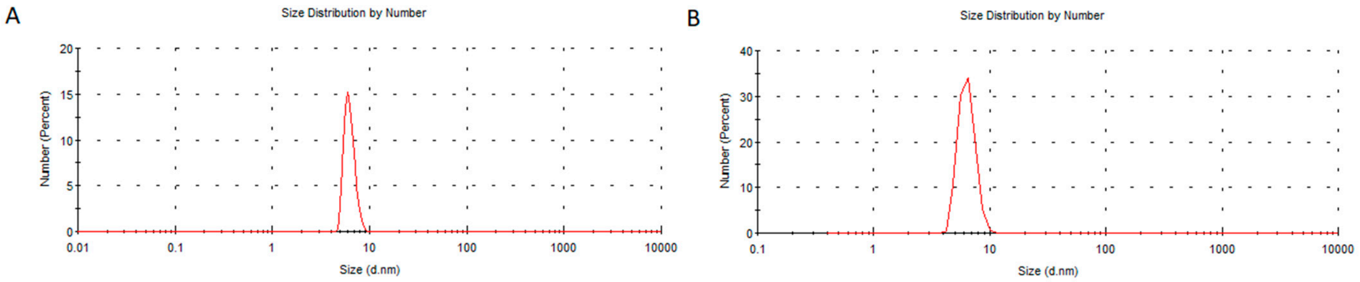

Figure S3. DLS size distribution of A – PFT-HSA; B – PFX-HSA by Number.

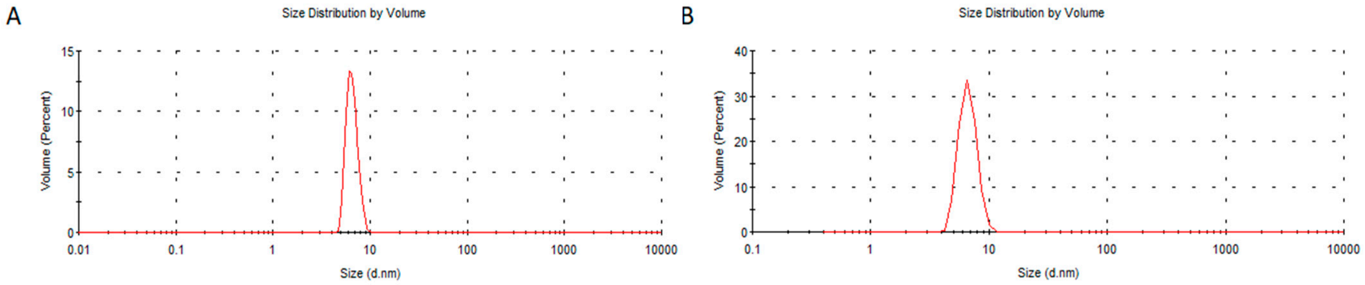

Figure S4. DLS size distribution of A – PFT-HSA; B – PFX-HSA by Volume.

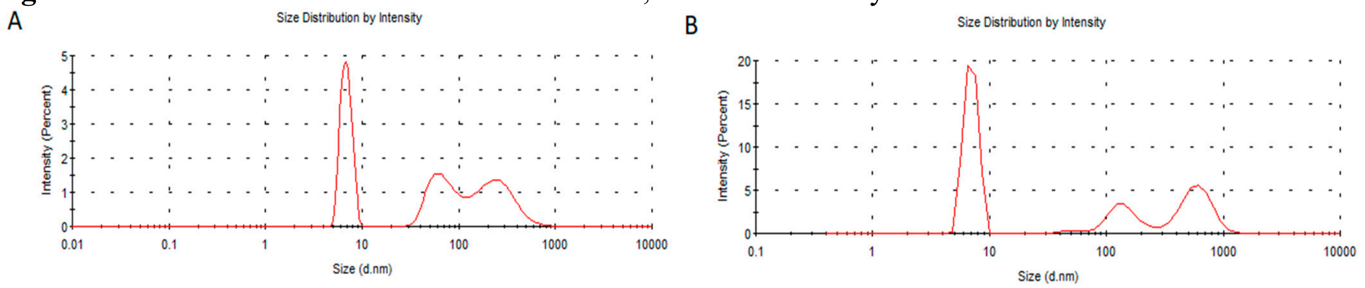

Figure S5. DLS size distribution of A – PFT-HSA; B – PFX-HSA by Intensity.

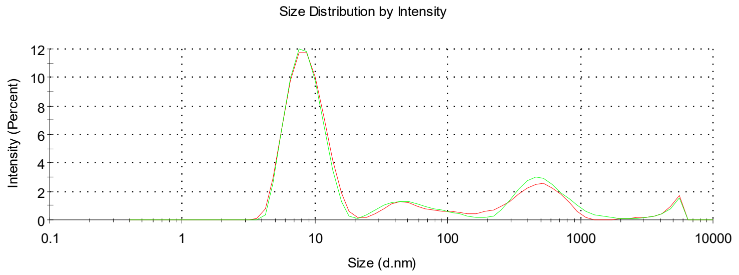

Figure S6. DLS size distribution of HSA by Intensity.

Flow cytometry. Cellular uptake of Cy5-labeled albumin conjugates was investigated by flow cytometry. A 2 × 10^5 T98G cells per well were seeded in 12-well plates in complete medium, treated with albumin conjugates, and incubated for 2 h. Afterward, the cells were washed with PBS three times, trypsinized using 0.25 % Trypsin-EDTA, and centrifuged at 1000 rpm for 5 min. The cells were fixed with 2% formaldehyde in PBS (20 min, 25 °C). The samples were measured in relative fluorescence units (RFU) on a FACS-Canto II flow cytometer (Becton Dickinson) using FACS Diva Software (BD Biosciences). Intact (non-stained) cells were used as a control.

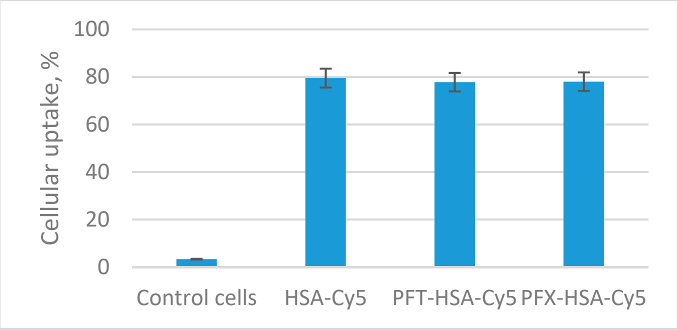

Figure S7. Cellular uptake of fluorescently labeled albumin conjugates.

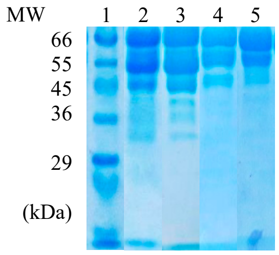

Figure S8. Typical albumin proteolysis SDS-PAGE data. Susceptibility of HSA and its conjugates to tryptic proteolysis (enzyme/substrate ratio 1:100). The bands in the gel lower 66.5 kDa (HSA MW) were marked as digested albumin. Quantitative data were obtained by digitizing the gel (SDS-PAGE) using GelPro Analyzer software (Media Cybernetics). SDS-PAGE (10%), pt. 6 h. The gel was stained with Coomassie Brilliant Blue. Line 1, MW markers (Sigma S8445); line 2, PFX-HSA; line 3, PFT-HSA; line 4, HSA; line 5, Hcy-HSA.
